# PnWRKY38-PnSUS1 axis regulates the biosynthesis of *Panax notoginseng* saponins

**DOI:** 10.1093/hr/uhag012

**Published:** 2026-01-13

**Authors:** Qin Chen, Xiaoying Wu, Yuan Qu, Na Li, Xiuming Cui, Feng Ge

**Affiliations:** Yunnan Key Laboratory of Sustainable Utilization of Panax notoginseng Resources, Faculty of Life Science and Technology, Kunming University of Science and Technology, Kunming 650500, China; State Key Laboratory for Quality Ensurance and Sustainable Use of Dao-di Herbs, Beijng 100700, China; Yunnan Key Laboratory of Sustainable Utilization of Panax notoginseng Resources, Faculty of Life Science and Technology, Kunming University of Science and Technology, Kunming 650500, China; State Key Laboratory for Quality Ensurance and Sustainable Use of Dao-di Herbs, Beijng 100700, China; Yunnan Key Laboratory of Sustainable Utilization of Panax notoginseng Resources, Faculty of Life Science and Technology, Kunming University of Science and Technology, Kunming 650500, China; State Key Laboratory for Quality Ensurance and Sustainable Use of Dao-di Herbs, Beijng 100700, China; Yunnan Key Laboratory of Sustainable Utilization of Panax notoginseng Resources, Faculty of Life Science and Technology, Kunming University of Science and Technology, Kunming 650500, China; State Key Laboratory for Quality Ensurance and Sustainable Use of Dao-di Herbs, Beijng 100700, China; Yunnan Key Laboratory of Sustainable Utilization of Panax notoginseng Resources, Faculty of Life Science and Technology, Kunming University of Science and Technology, Kunming 650500, China; State Key Laboratory for Quality Ensurance and Sustainable Use of Dao-di Herbs, Beijng 100700, China; Yunnan Key Laboratory of Sustainable Utilization of Panax notoginseng Resources, Faculty of Life Science and Technology, Kunming University of Science and Technology, Kunming 650500, China; State Key Laboratory for Quality Ensurance and Sustainable Use of Dao-di Herbs, Beijng 100700, China

## Abstract

Sucrose synthase (SUS) is a pivotal enzyme bridging primary carbon metabolism and secondary biosynthesis in plants. In *Panax notoginseng*, we demonstrate that sucrose synthase 1 (PnSUS1) serves as a metabolic bottleneck for saponin glycosylation by supplying UDP-glucose. PnWRKY38 was identified as a WRKY transcription factor whose expression correlated with both PnSUS1 and saponin accumulation. Overexpression of *PnWRKY38* could up-regulate *PnSUS1* expression by 3.5-fold, increase SUS enzyme activity by 2.8-fold, and elevate UDP-glucose pools by 68%. Consequently, the total content of ginsenosides Rg1, Rb1, and Rd rose by 2.1–2.4-fold. Conversely, PnSUS1 or PnWRKY38 suppression reduced UDP-glucose available and saponin biosynthesis by >50%. Y1H and luciferase assays indicated that PnWRKY38 directly activated *PnSUS1* expression by binding to W-box motifs in its promoter. These results not only illustrate the specific function that PnSUS1 executes in UDPG biosynthesis but also reveal a new WRKY transcriptional regulatory module regulating notoginsenosides production.

## Introduction

*Panax notoginseng* (Burk.) F.H. Chen, a valued member of the Araliaceae family, is a perennial herb with a rich history in traditional Chinese medicine spanning centuries [1]. Commonly known as Sanqi or Tianqi, its dried rhizomes and roots are prized for their remarkable therapeutic properties, including hemostatic, anti-inflammatory, neuroprotective, and cardiovascular-protective effects [2, 3]. The primary bioactive constituents responsible for these pharmacological activities are the *P. notoginseng* saponins (PNS), a class of triterpenoid saponins [4]. PNS mainly include ginsenosides Rg1, Re Rb1, Rd, and notoginsenoside R1, whose accumulation levels directly determine the quality and clinical efficacy of *P. notoginseng* medicinal materials [5]. Consequently, there is a substantial commercial demand for high-quality *P. notoginseng*. However, challenges such as long growth cycles, specific environmental requirements, and the decline in active ingredient content due to continuous cropping obstacles severely limit its large-scale production and consistent quality [6–8]. Therefore, elucidating the molecular mechanisms governing PNS biosynthesis is of paramount importance for developing strategies to enhance saponin yield through metabolic engineering or molecular breeding [9].

The biosynthesis of the triterpenoid saponin backbone in plants is a complex and energy-intensive process. It begins with the universal isoprenoid precursors, isopentenyl pyrophosphate (IPP), and dimethylallyl pyrophosphate (DMAPP) [10]. These precursors are generated through two distinct pathways, the mevalonate (MVA) pathway in the cytoplasm and the methylerythritol phosphate (MEP) pathway in the plastids [11]. The IPP and DMAPP are converted into 2,3-oxidosqualene by a series of enzymes, the final common precursor for all triterpenoids [12–14]. This backbone then undergoes cyclization by oxidosqualene cyclases (OSCs) to form various triterpenoid precursors. These precursors are subsequently modified by a series of downstream enzymes, including cytochrome P450 monooxygenases (CYP450s) and UDP-glycosyltransferases (UGTs), which results in the vast structural diversity of ginsenosides [15, 16]. However, the formation of the saponin skeleton is only the first step. Subsequent glycosylation modification is key to its diverse biological activities, and this process is highly dependent on an adequate supply of activated sugar donors [17].

Uridine diphosphate glucose (UDP-Glc) plays an indispensable and pivotal role in the biosynthesis of ginsenosides, serving as the primary activated sugar donor for the final and functionally critical glycosylation steps [18]. The core triterpenoid aglycones, such as protopanaxadiol (PPD) and protopanaxatriol (PPT), are biologically inert or have low activity and poor solubility [19]. The transformation of these precursors into a diverse array of pharmacologically active ginsenosides is entirely dependent on the sequential attachment of glucose moieties, a process catalyzed by UGTs [10]. These enzymes specifically utilize UDP-Glc to transfer glucose to the hydroxyl groups on the sapogenin backbone (Fig. S1). This glycosylation is fundamental for two reasons: firstly, it generates the vast structural diversity of the ginsenoside family (e.g. Rb1, Rg1, Re), as the number and position of sugar attachments define each unique compound [20]. Secondly, and more importantly, the glycone moieties are crucial determinants of the ginsenosides’ physicochemical properties and biological activities [21]. They enhance water solubility, which is critical for absorption and bioavailability, and modulate the molecule’s ability to interact with specific cellular targets, thereby dictating its therapeutic efficacy [22]. Consequently, the cellular pool and availability of UDP-Glc, which bridges primary carbohydrate metabolism with secondary metabolite production, act as a critical metabolic flux point and a potential rate-limiting factor for the overall accumulation of valuable ginsenosides in *Panax* species [23].

Sucrose synthase (SUS) is a pivotal enzyme in carbohydrate metabolism, catalyzing the reversible conversion of sucrose and UDP into UDP-glucose and fructose [24]. In addition to its role in sugar allocation, recent studies have implicated SUS in supplying carbon skeletons and energy for the biosynthesis of secondary metabolites [25]. It serves as a common sugar-donor substrate for glucosyltransferases, facilitating the production of various active glycosides such as nothofagin, ginsenosides, and salidroside [26]. Furthermore, UDP-Glc can act as a precursor for the synthesis of other sugar donors including uridine 5′-diphosphate-galactose (UDP-Gal) and UDP-glucuronic acid—essential substrates for the biosynthesis of human milk oligosaccharides, hyaluronic acid, and other bioactive oligosaccharides [27]. In *P. notoginseng*, sucrose is a major photosynthetic product transported to roots where saponins are synthesized [28]. Therefore, SUS may serve as a critical link between primary carbohydrate metabolism and saponin accumulation.

There are various ways to regulate glycosylation modification, among which transcriptional regulation is one of the most important ones. Transcription factors (TFs) are pivotal regulators that control gene expression at the transcriptional level, thereby orchestrating various aspects of plant growth, development, and metabolism [29]. The WRKY family, one of the largest families of TFs in plants, is characterized by a highly conserved WRKYGQK DNA-binding domain [30]. WRKY TFs are known to play crucial roles in diverse biological processes, including responses to biotic and abiotic stresses, senescence, and the regulation of secondary metabolite biosynthesis [31, 32]. For instance, in *Artemisia annua*, AaWRKY1 activates the expression of the artemisinin biosynthesis gene *ADS* [33]. In *Salvia miltiorrhiza*, SmWRKY2 has been shown to regulate the biosynthesis of tanshinone [34]. Recent studies in *Panax* species have begun to uncover the role of WRKY TFs in saponin biosynthesis; however, the functions of most members of this large family in *P. notoginseng* have yet to be characterized [35, 36]. Although some WRKY transcription factors have been reported to be involved in regulating key enzyme genes in the saponin skeleton synthesis pathway (such as DS and SS), to date, no studies have focused on transcription factors that regulate the supply of upstream sugar donors (such as UDP-Glc), which may be the ‘master valve’ of the entire metabolic flux [37]. Elucidating this upstream regulatory mechanism is crucial for systematically enhancing saponin yield.

In this study, we identified a novel WRKY transcription factor PnWRKY38 from *P. notoginseng* and demonstrated that PnWRKY38 played a positive role in the regulation of PNS biosynthesis. PnWRKY38 could directly bind to the promoter of the sucrose synthase gene (PnSUS1), enhancing its expression and thereby increasing the availability of UDP-glucose, a key sugar donor for ginsenoside glycosylation. Our findings reveal a novel regulatory module in which PnWRKY38 promotes the biosynthesis of saponins in *P. notoginseng* by transcriptionally activating the expression of SUS, providing a new insight into the metabolic engineering of natural products in plants.

## Results

### Identification and characterization of a SUS genes from the *P. notoginseng* genome

**Figure 1:**
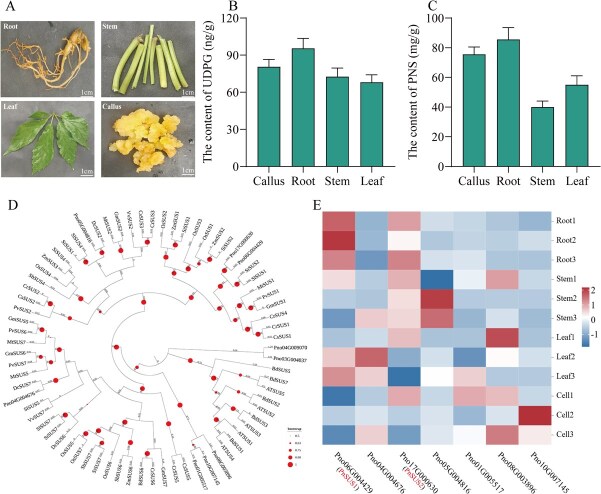
Identification of *PnSUS* from *P. notoginseng* genome. (A) Different tissues of *P. notoginseng*. (B) Content of UDPG in different tissues. (C) Content of PNS in different tissues. (D) Phylogenetic analysis of PnSUS proteins from *P. notoginseng* and other plants. The phylogenetic tree was constructed using MEGA-7, based on the multiple sequence alignment performed by ClustalX and employing the neighbour-joining method with a rooted approach. The size of the orange circles on the branches indicates the bootstrap values derived from 1000 bootstrap replicates, with a cut-off threshold set below 50%. Species abbreviations: At, *Arabidopsis thaliana*; Pn, *Panax notoginseng*; Cr, *Capsella rubella*; Br, *Brassica rapa*; Gm, *Glycine max*; Pv, *Phaseolus vulgaris*; Mt, *Medicago truncatula*; Vv, *Vitis vinifera*; Sl, *Solanum lycopersicum*; St, *Solanum tuberosum*; Dc, *Daucus carota*; Si, *Setaria italica*; Zm, *Zea mays*; Sb, *Sorghum bicolor*; Bd, *Brachypodium distachyon*; Os, *Oryza sativa*; Cs, *Camelina sativa*. (E) The expression analysis of seven PnSUS genes at different location of *P. notoginseng.*

To identify the potential SUS genes involved in notoginsenosides production, we searched for the genes annotated to encode sucrose synthetase in the *P. notoginsng* genome database (https://www.citrusgenomedb.org/) and seven putative SUS genes were identified. Chromosomal localization analysis revealed that seven genes were distributed across seven chromosomes. Collinearity analysis with *Arabidopsis thaliana* SUS genes identified four AtSUS genes and seven PnSUS genes forming four conserved gene pairs (Fig. S2B), suggesting gene duplication as a potential driver for the expansion and evolution of SUS genes in the *P. notoginseng* genome. Phylogenetic analysis showed that these seven proteins clustered into three distinct subfamilies (Fig. 1D). Among these, two SUS genes, PnSUS1 (Pn006G004429) and PnSUS2 (Pno17G000630), exhibited the highest expression levels in roots and the lowest in leaves (Fig. 1E). This distribution pattern aligns with the accumulation of notoginsenosides in *P. notoginseng*  **(**Fig. 1C) and mirrors the highest UDP-glucose (UDPG) content observed in roots compared to stems and leaves (Fig. 1B). These findings indicate that PnSUS1 and PnSUS2 may participate in the biosynthesis of notoginsenosides.

### In vitro enzyme assays of recombinant PnSUS protein

**Figure 2:**
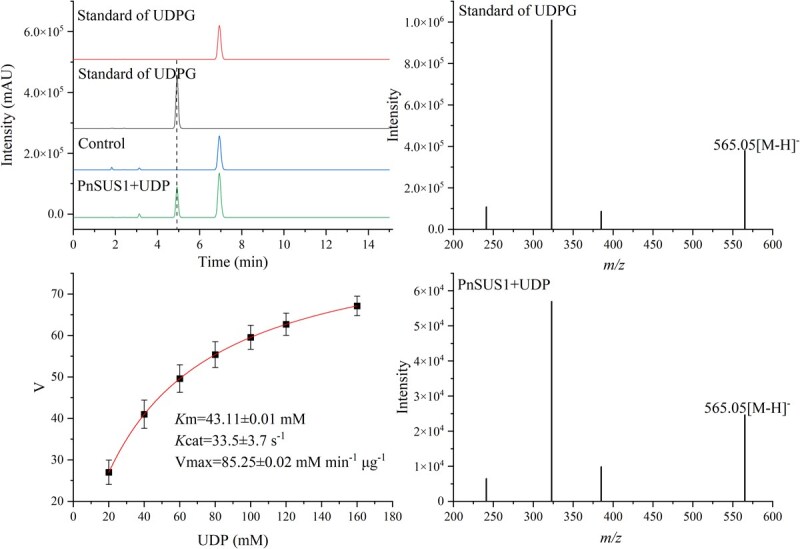
*In vitro* enzymatic assays of the recombinant PnSUS1. (A) HPLC analyses of reaction products. (B) The mass spectra of UDPG standard and reaction products produced by incubation with PnSUS1. (C) Enzyme kinetic analysis of PNSUS1. (D) Molecular docking of PNSUS1 to UDP.

Enzymatic activity assays of PnSUS1 and PnSUS2 were performed. The results demonstrated that the target product UDP-glucose (UDPG) was successfully detected in the reaction products of PnSUS1, confirming its ability to catalyze the glycosylation of UDP to form UDPG (Fig. 2). In contrast, no UDPG was detected in the reaction products of PnSUS2 (Fig. S3). Molecular docking analysis revealed a binding energy of −9.5 kcal/mol for the interaction between PnSUS1 and UDP, further validating the efficient binding of PnSUS1 to its substrate UDP (Fig. S4). Enzyme kinetic analysis was conducted to characterize the catalytic activity of PnSUS1. As shown in Fig. 2, PnSUS1 exhibited a *V*max of 85.25 ± 0.02 mmol min^−1^ μg^−1^, *K*m value of 43.11 ± 0.01 mM, and the *k*cat of 33.5 ± 3.7 s^−1^.

### PnSUS1 positively regulated biosynthesis of saponins in *P. notoginseng*

**Figure 3:**
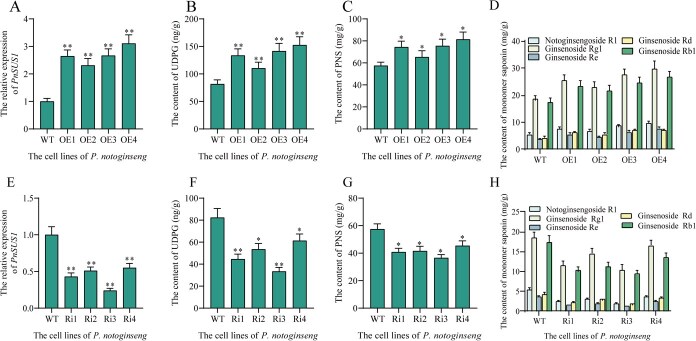
Overexpression and RNA interference of *PnSUS1* in *P. notoginseng*. The experiment was conducted three times, and error bars reflect the standard deviation (SD) from three independent biological replicates. Asterisks show statistically significant differences relative to WT. Statistical analysis was performed by one-way ANOVA with Duncan’s multiple range tests to separate means. ^**^P < 0.01, ^*^P < 0.05.

In vitro enzyme activity assays demonstrated that PnSUS1 catalyzes the conversion of UDP to UDPG. Homologous overexpression and RNAi manipulations were carried out to confirm the notoginsengosides accumulation-enhancing function of PnSUS1 in *P. notoginseng*. In *PnSUS1*-overexpressing *P. notoginseng* cell lines, the expression level of PnSUS1 was significantly increased, confirming the successful generation of these transgenic lines. At the same time, the expression levels of UGTs related to saponin synthesis also increased significantly (Fig. S5A). The UDPG content was markedly elevated in the overexpression lines, reaching 1.81-fold higher in the OE4 line compared to the wild type (WT). This increase in UDPG significantly boosted the levels of total *P. notoginseng* saponins and individual saponin monomers. Specifically, the total saponin content in the OE4 line was 1.24 times higher than in WT cells. Furthermore, the contents of ginsenoside Rd, ginsenoside Rb1, ginsenoside Rg1, ginsenoside Re, and notoginsenoside R1 in the overexpression lines were 1.7-fold, 1.5-fold, 1.6-fold, 2.1-fold, and 1.7-fold higher, respectively, than in the WT cells. Conversely, in the RNAi-mediated *PnSUS1*-silenced cell lines, the expression level of *PnSUS1* was reduced to varying degrees across four positive lines (Fig. 3E). Similarly, the expression levels of UGTs associated with saponin synthesis were also significantly reduced (Fig. S5B). RNAi of *PnSUS1* gene expression led to decreased UDPG content (Fig. 3F), accompanied by a significant reduction in both total saponins and individual saponin monomers. These results provide direct evidence that PnSUS1 participates in UDPG biosynthesis and effectively promotes the production of *P. notoginseng* saponins (Fig. 3H).

### Screening of potential transcription factors activating PnSUS1 transcription

**Figure 4:**
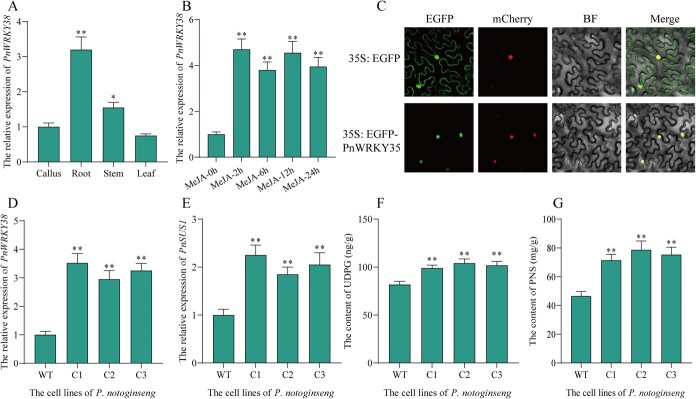
Characterization and overexpression of *PnWRKY38*. (A) The expression level of *PnWRKY38* in different tissues. (B) The expression level of PnWRKY38 in the roots of *P. notoginseng* with or without MeJA treatment. (C) Subcellular localization of *PnWRKY38*. mCherry indicates a nuclear marker with nuclear localization signal (NLS) fused with mCherry. (D–G) The expression levels of related genes, UDPG content and total content of saponins in the *P. notoginseng* cell line overexpressed *PnWRKY38*. C1–C3, *P. notoginseng* cell lines overexpressed *PnWRKY38*. The experiment was conducted three times, and error bars reflect the SD from three independent biological replicates. Asterisks show statistically significant differences relative to WT. Statistical analysis was performed by one-way ANOVA with Duncan’s multiple range tests to separate means. ^**^P < 0.01.

A 2000-bp fragment of the promotor region of *PnSUS1* was cloned upstream of the ATG codon. PlantTFDB was analyzed the promoter of *PnSUS1* and found that it contained three W-box elements, suggesting that the expression of *PnSUS1* may be regulated by the WRKY family. A total of 9 TFs were screened by yeast one-hybrid (Y1H) library screening with *PnSUS1* promoter as bait. Fortunately, we identified one WRKY transcription factor (PnWRKY38) that has an activating effect on the promoter of *PnSUS1* (Fig. S6). To investigate the tissue-specific expression profile of PnWRKY38 in *P. notoginseng*, RT-qPCR analysis was performed. The results revealed that PnWRKY38 was predominantly expressed in subterranean tissues, with the highest transcript abundance observed in the root. Conversely, the lowest levels of expression were detected in the leaf (Fig. 4A). Furthermore, the expression of PnWRKY38 was found to be strongly induced by the plant hormone Methyl Jasmonate (Fig. 4B). Given that transcription factors typically function within the nucleus, we proceeded to determine the subcellular localization of the PnWRKY38 protein. For this purpose, a PnWRKY38-EGFP fusion protein was transiently expressed in the epidermal cells of *Nicotiana benthamiana* leaves. Confocal microscopy revealed that the PnWRKY38-EGFP fusion protein was exclusively localized to the nucleus, where it co-localized with a nuclear marker. In contrast, the EGFP control protein was distributed throughout the entire cell, including the cytoplasm and nucleus (Fig. 4C). This specific nuclear localization provides strong support for the role of PnWRKY38 as a transcriptional regulator.

To further investigate the function of PnWRKY38 in the regulation of saponin biosynthesis, the transgenic *P. notoginseng* cell overexpressing *PnWRKY38* was constructed. The successful overexpression of *PnWRKY38* was confirmed in three independent transgenic lines (C1, C2, and C3), which exhibited significantly elevated transcript levels compared to the WT control (Fig. 4D). In the *P. notoginseng* cell lines of overexpressing *PnWRKY38*, the expression levels of UGTs related to saponin synthesis also increased. However, the Dual-luciferase assay (Luciferase, LUC) indicates that PnWRKY38 does not directly act on the promoter of UGTs (Fig. S7). The reason might that the expression of PnWRKY38 promotes the expression of *PnSUS1*, which in turn promotes the expression of related UGTs. Concurrently, the transcript levels of the *PnSUS1* were significantly upregulated in the *PnWRKY38*-overexpressing lines (Fig. 4E). Consistent with these changes in gene expression, the content of UDPG, the sugar donor for glycosylation, was significantly increased (Fig. 4F). Ultimately, this coordinated upregulation led to a significant elevation in the total saponin content in the transgenic cell (Fig. 4G).

### PnWRKY38 directly bind to the PnSUS1 promoter

**Figure 5:**
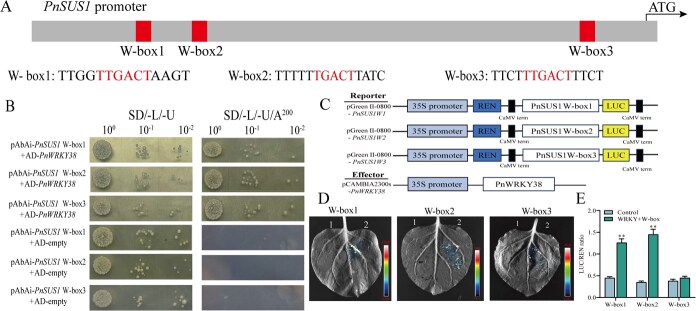
PnWRKY38 directly binds to and activates the promoter of *PnSUS1*. (A) W-boxes in PnSUS1 promoter. (B) Y1H assay showing PnWRKY38 binding to W-boxes of PnSUS1 promoter. (C) Schematic diagram of dual-LUC vectors and dual-LUC confirming the activation of PnWRKY38 on PnSUS1. (D) PnWRKY38 and W-boxes of Luciferase intensity image in *N. benthamiana* leaves. 1, pGreen0800 + pCAMBIA2300- PnWRKY38. 2, pGreen0800-W-box1/2/3 + pCAMBIA2300- PnWRKY38. (E) Regulatory effects of PnWRKY38 on the promoter of PnSUS1. The experiment was conducted three times, and error bars reflect the SD from three independent biological replicates. Statistical significance was determined using a Student’s *t*-test, with ^**^P < 0.01 indicating significance.

According to the analysis by Plant TFDB, the promoter of PnSUS1 contains three binding regions of W-box, Within the promoter region, a total of 3 W-boxes were identified, which are W-box1 (TTGACC), W-box2 (TTGACC), and W-box3(TTGACC) (Fig. 5A). Given that WRKY transcription factors are established regulators of specialized metabolite biosynthesis, we designed Y1H and dual-luciferase assays to to determinewhether PnWRKY38 directly regulates the expression of PnSUS1. Indeed, the Y1H results revealed a direct physical interaction, showing that the PnWRKY38 protein specifically binds to the W-box1, W-box2, and W-box3 motifs in the promoter region of PnSUS1 (Fig. 5B). Subsequently, the Dual-LUC was used to determine the strength of the promoter’s effect. PnWRKY38 could activate the expression of W-box1 and W-box2 regions with increment of 2.8-fold and 4.14-fold, respectively, However, it had no significant effect on the activity of W-box3 (Fig. 5E). These results demonstrate that PnWRKY38 mainly activates the expression of PnSUS1 by activating the activities of W-box1 and W-box2 in *PnSUS1* promoter.

### Effects of co-overexpressing *PnWRKY38 and PnSUS1* in *P. notoginseng*

**Figure 6:**
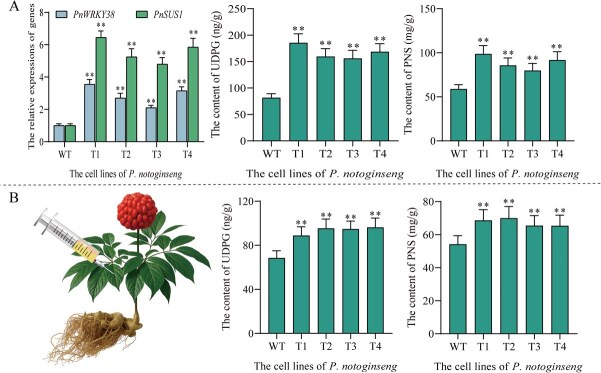
Co-overexpression of *PnSUS1* and PnWRKY38 in *P. notoginseng*. (A) the content of UDPG and PNS of co-expressed by *PnSUS1* and *PnWRKY38* in the *P. notoginseng* callus. (B) The content of UDPG and PNS of co-expressed by *PnSUS1* and *PnWRKY38* in the *P. notoginseng* leaves. The experiment was conducted three times, and error bars reflect the SD from three independent biological replicates. Asterisks show statistically significant differences relative to WT. Statistical analysis was performed by one-way ANOVA with Duncan’s multiple range tests to separate means. ^**^P < 0.01, ^*^P < 0.05.

To explore the effect of *PnWRKY38* and *PnSUS1* co-expression on the biosynthesis of *P. notoginseng* saponins, *PnWRKY38* and *PnSUS1* were overexpressed in *P. notoginseng* cells. In the *P. notoginseng* cell line overexpressing *PnSUS1*, the expression of *PnSUS1* and *PnWRKY38* was significantly increased, and the expression of PnSUS1 was 2.43-fold higher than that of Pnsus1 overexpression alone (T1 line), while the expression of *PnWRKY38* was basically unchanged compared with that of *PnSUS1* overexpression alone. In the co-expressed *P. notoginseng* cell line, the amount of UDPG was 2.3-fold higher than that of WT and 1.4-fold higher than that of PnSUS1 alone. The increase of UDPG content also significantly increased the total saponins content in *P. notoginseng* cells. In the *P. notoginseng* cell lines co-expressed with UDPG, the total saponins content was 1.68-fold higher than that in normal cells and 1.3-fold higher than that in PnSUS1 alone (Fig. 6A). Correspondingly, the contents of monomeric saponins ginsenoside Rd, Rb1, Rg1, Re, and notoginsenoside R1 in *P. notoginseng* cell were also significantly increased **(**Fig. S8**)**. In the overexpressed *P. notoginseng* leaves, the expression level of UDPG and the content of PNS also significantly increased (Fig. 6B).

## Discussion

**Figure 7:**
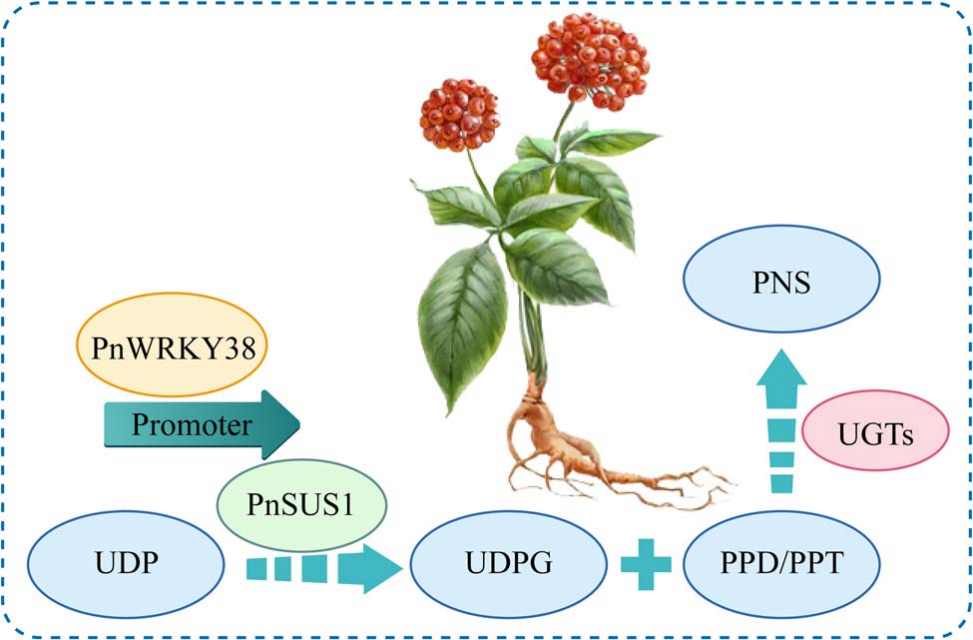
A proposed model for the molecular mechanism of PnWRKY38-mediated regulation of PNS biosynthesis. In this model, the WRKY transcription factor PnWRKY38 functions as an upstream positive regulator. It directly binds to the W-box cis-acting elements in the promoter region of the SUS gene, PnSUS1, to activate its transcriptional expression. The enhanced expression of *PnSUS1* leads to increased PnSUS1 enzyme activity, which catalyzes the efficient conversion of UDP to UDP-glucose (UDPG). The expansion of the intracellular UDPG pool provides an ample supply of sugar donors for the saponin glycosylation process. Ultimately, catalyzed by downstream UDP-glycosyltransferases (UGTs), UDPG is used to transfer glucose moieties onto sapogenin precursors (such as PPD and PPT), significantly promoting the biosynthesis and accumulation of various saponin monomers and total PNS in *P. notoginseng.*

This study successfully elucidates a key regulatory module in *P. notoginseng* that links primary carbon metabolism with the biosynthesis of secondary saponins, as shown in (Fig. 7). We have for the first time identified and functionally validated a WRKY transcription factor, PnWRKY38, which regulates saponin biosynthesis by directly activating the transcription of the sucrose synthase gene, *PnSUS1*. This transcription factor significantly enhances the cellular supply of UDP-glucose, a precursor for saponin glycosylation. This finding not only clarifies a previously uncharacterized regulatory mechanism but also provides new molecular targets and a theoretical framework for improving the economic and medicinal value of this important medicinal plant through metabolic engineering.

Due to its natural abundance and low cost, sucrose is an ideal substrate for the enzymatic production of UDP-Glc. This reaction is catalyzed by SUS, which facilitates the transglycosylation from sucrose to UDP. However, the industrial viability of this bioprocess is frequently hindered by the inherent limitations of native SUS, namely their low catalytic activity and poor thermostability. These deficiencies present significant challenges, such as requiring prolonged reaction times and resulting in low product yields, which impede the cost-effective generation of UDP-Glc [38]. The resulting high production cost has, in turn, limited the broader application of UDP-Glc. Therefore, there is a compelling demand to engineer improved SUS variants with enhanced catalytic efficiency and stability to enable more robust and economical bioprocesses. SUS acts as a bridge in plants, channeling primary carbon metabolites toward the synthesis of secondary metabolites [39]. In this study, we identified seven putative *PnSUS* genes from the *P. notoginseng* genome, among which *PnSUS1* and *PnSUS2* exhibited the highest expression levels in the roots, the primary site of saponin accumulation. Notably, in vitro enzyme assays revealed that only PnSUS1 could effectively catalyze the formation of UDPG, whereas PnSUS2 showed no such activity, indicating significant functional divergence among *PnSUS* gene family members. Compared with other types of SUS catalysis, PnSUS1 exhibits higher catalytic efficiency. This might be because a large amount of UDPG is required during the biosynthesis of ginsenosides [26]. The highly efficient synthesis method for SUS has been discovered, which also provides a possibility for the synthesis of secondary metabolites that require glycosylation. Through overexpression and RNAi-mediated silencing experiments of *PnSUS1* in *P. notoginseng* cells, we confirmed its bottleneck role in the saponin biosynthesis pathway. Overexpression of *PnSUS1* not only significantly increased UDPG content but also elevated total saponin accumulation to 1.24-fold higher than the WT, with a substantial increase in several key individual saponins such as ginsenosides Rd, Rb1, and Rg1. Conversely, suppression of *PnSUS1* expression led to a reduction in UDPG and saponin content by over 50%. This direct evidence strongly demonstrates that PnSUS1 is the core enzyme responsible for supplying the key sugar donor, UDPG, for the saponin biosynthesis pathway in *P. notoginseng*.

Transcription factors are core components of the regulatory networks for plant secondary metabolism, and previous studies have shown that WRKY family transcription factors regulate the biosynthesis of terpenoids and phenolics in various medicinal plants [31]. In this study, through transcriptome co-expression analysis and a Y1H library screening, we successfully identified a new WRKY transcription factor, PnWRKY38. Multiple lines of evidence indicate that PnWRKY38 is a direct positive regulator of *PnSUS1* transcription. First, the tissue expression pattern of *PnWRKY38* is highly consistent with that of *PnSUS1*, with both being highly expressed in the roots. Second, subcellular localization experiments confirmed that PnWRKY38 is a nuclear protein, which is consistent with its function as a transcription factor. The most direct evidence comes from a series of molecular interaction assays: Y1H and dual-luciferase reporter assays all consistently confirmed that PnWRKY38 specifically binds to the W-box cis-acting elements in the promoter region of *PnSUS1* and significantly activates its transcriptional activity, with up to a 4.2-fold induction. Correspondingly, functional validation experiments demonstrated that overexpression of *PnWRKY38* could upregulate *PnSUS1* expression by 3.5-fold, increase SUS enzyme activity and the UDPG pool by 2.8-fold and 68%, respectively, and ultimately boost the total saponin content by 2.1- to 2.4-fold.

This study clearly delineates the PnWRKY38-PnSUS1 regulatory mechanism, which not only provides a new perspective for understanding *P. notoginseng* saponin synthesis but also offers important insights for metabolic engineering strategies. In the co-expression experiment of *PnSUS1* and *PnWRKY38*, we observed a significant synergistic effect. Compared to cell lines overexpressing *PnSUS1* alone, the UDPG and total saponin contents in the co-expressing cell lines were further increased by 1.4-fold and 1.3-fold, respectively. This result suggests that merely increasing the expression of a single enzyme in a biosynthetic pathway may be limited by its upstream transcriptional regulation, manipulating key transcription factors (like PnWRKY38) to systematically ‘unlock’ the entire metabolic flux could be a more efficient strategy for enhancing production. This finding also further establishes SUS as one of the primary targets for enhancing triterpenoid saponin production in medicinal plants.

In conclusion, this study has fully elucidated a complete regulatory chain from transcription factor regulation to enzymatic reaction and final product accumulation (PnWRKY38 → *PnSUS1* gene → PnSUS1 enzyme → UDPG → saponins), greatly deepening our understanding of the complex metabolic network regulation in this important medicinal plant, especially given that its regulatory mechanisms remain incompletely understood. Future research could further explore the upstream signaling pathways that regulate PnWRKY38 expression (e.g. how it responds to developmental or environmental factors), and whether other transcription factors act synergistically with PnWRKY38 to form a more intricate regulatory network. Furthermore, applying this effective strategy, validated in cell culture, to the genetic improvement of whole *P. notoginseng* plants could lead to the breeding of superior varieties with significantly enhanced saponin content, thereby meeting the growing demands of the global pharmaceutical market.

## Materials and methods

### Plant materials and chemicals

Healthy roots of *P. notoginseng* were selected as explants. After initial surface sterilization with 70%–75% ethanol, the explants were thoroughly sterilized with 2%–5% sodium hypochlorite solution and then rinsed thoroughly with sterile distilled water. The aseptic explants were inoculated onto Murashige and Skoog (MS) solid medium supplemented with 1 mg l^−1^ 2,4-D and 1.0 mg l^−1^ kinetin, containing 0.7%–0.8% agar (pH adjusted to 5.6–5.8). Cultures were maintained under dark conditions at 22°C–25°C, and friable, light-yellow callus tissues formed within ~3–4 weeks. Ginsenoside standards (all ginsenosides present in the study) were purchased from Weikeqi Biological Technology (Chengdu, Sichuan, China), with a purity of 98%. The standard samples of UDP and UDPG were all purchased from Yuanye Bio-Technology (Shanghai, China).

### Mining SUSs from *P. notoginseng*

The reference genome of *P. notoginseng* was retrieved from the National Genomics Data Center (NGDC, https://ngdc.cncb.ac.cn/). To identify the SUS gene family, a Hidden Markov Model (HMM) profile for the SUS domain (PF00862) was obtained from the Pfam database [40]. This profile was then utilized to screen the *P. notoginseng* genome for potential SUS family members using HMMER. The conserved functional domains of the resulting candidate genes were verified using the Simple Modular Architecture Research Tool (SMART), and sequences with incomplete domains were subsequently excluded from further analysis. Finally, a maximum-likelihood phylogenetic tree was constructed using aligned full-length protein sequences of *P. notoginseng* SUSs, *A. thaliana sucrose* SUSs, *Capsella rubella* SUSs, *Phaseolus vulgaris* SUSs, *Medicago truncatula* SUSs, *Vitis vinifera* SUSs, *Solanum lycopersicum* SUSs, *Solanum tuberosum* SUSs, *Daucus carota* SUSs, *Setaria italica* SUSs, *Zea mays* SUSs, *Sorghum bicolor* SUSs, *Brachypodium distachyon* SUSs, *Oryza sativa* SUSs, *Camelina sativa* SUSs, and *Glycine max* SUSs via MEGA7, enabling evolutionary relationship inference.

### RNA extraction and gene expression analysis

Total RNA of *P. notoginseng* was isolated using the Trizol reagent (Invitrogen, USA), and subsequently served as a template for first-strand cDNA synthesis with the Reverse Transcription System (Promega, USA), adhering to the manufacturer’s protocols. Quantitative real-time PCR (RT-qPCR) was performed on a CFX Connect Real-Time PCR Detection System (Bio-Rad, USA) with the SYBR Green PCR Master Mix (Vazyme Biotech, China). Gene expression levels were normalized to the endogenous reference gene, *β-actin*, and relative expression was calculated using the 2^−ΔΔCt^ method. The analysis was conducted with three biological and three technical replicates for each sample. All primer sequences used in this study are listed in Table S1.

### Genes isolation, promoter cloning, and plasmid construction

To begin, genomic DNA was extracted from *P. notoginseng* roots using the EZ-10 Spin Column Plant Genomic DNA Purification Kit (Sangon Biotech, Shanghai, China). This gDNA was used as a template to amplify the 2.0-kb promoter region of *PnSUS1*. Concurrently, the full-length coding sequences (CDS) for all genes under investigation were amplified from a *P. notoginseng* cDNA library. All amplicons were cloned into the pMD18-T vector (Takara, Dalian, China) and verified by Sanger sequencing.

For the expression of recombinant proteins, the CDS of *PnSUS1* and *PnSUS2* were amplified with flanking *Kpn I* and *BamH* I restriction sites and ligated into the pCOLD-TF expression vector. These constructs were then transformed into the *Escherichia coli* Rosetta (DE3) strain.

For in vivo analyses, several constructs were prepared. For overexpression, the CDS of *PnSUS1* and *PnWRKY38* were cloned into the pCAMBIA1300 binary vector. For RNA interference (RNAi), a 300-bp fragment of the *PnSUS1* CDS was inserted into the pHellsgate vector [41]. These plant expression constructs were subsequently transferred into *Agrobacterium tumefaciens* strain LBA4404.

To determine subcellular localization, the ORF of *PnWRKY38* was cloned into the pCAMBIA1300-eGFP vector, creating an eGFP fusion construct. This plasmid was introduced into *A. tumefaciens* strain GV3101 by electroporation.

For Yeast One-Hybrid (Y1H) assays, the PnSUS1 promoter was ligated into the pAbAi bait vector, and the ORF of PnWRKY38 was inserted into the pGADT7 prey vector. For dual-luciferase assays, the PnSUS1 promoter was cloned into the pGreenII 0800-LUC reporter vector.

A comprehensive list of primers used for all plasmid constructions is provided in Table S1.

### Protein expression in *E. coli* and PnSUS1 activity assay

Transformed *E. coli* cells harboring the expression constructs were cultured in 500 ml of Luria-Bertani (LB) medium at 37°C with agitation until the optical density at 600 nm (OD_600_) reached 0.6. Protein expression was then induced by the addition of isopropyl-β-D-thiogalactopyranoside (IPTG) to a final concentration of 0.5 mM, followed by incubation for 17 h at 16°C. Cells were harvested by centrifugation (5000 × *g*, 10 min, 4°C) and the resulting pellet was resuspended in 5 ml of phosphate-buffered saline (PBS). The resuspended cells were lysed by sonication on ice and cell debris was removed by centrifugation (12 000 × *g*, 15 min, 4°C). The clarified supernatant, representing the crude enzyme extract, was collected for subsequent assays.

The enzymatic activity of PnSUS was measured in a 500 μl reaction volume containing 50 mM Tris–HCl (pH 6.8), 5 mM uridine diphosphate (UDP), 50 mM sucrose, and 164 μl of the crude enzyme extract. The reaction was incubated for 1 h at 37°C and subsequently terminated by heating in a boiling water bath for 5 min. Precipitated proteins were removed by centrifugation, and the supernatant was collected for analysis. To determine the kinetic parameters of the purified PnSUS1 enzyme, reactions were conducted in a 500 μl mixture containing 50 mM Tris–HCl (pH 6.8), 5 mM UDP, 20 μg of purified recombinant protein, and varying concentrations of sucrose (0, 50, 100, 150, 200, and 250 mM). The reactions were incubated and terminated under the same conditions described for the standard activity assay.

The quantification of reaction products was performed on a Shimadzu LC-20 AD High-Performance Liquid Chromatography (HPLC) system, fitted with an Ultimate Plus-C18 column (4.6 × 150 mm, 5 μm particle size). Chromatographic separation was achieved under isocratic conditions at a column temperature of 30°C. The mobile phase consisted of a buffer and acetonitrile mixture (87:13, v/v), delivered at a constant flow rate of 1.0 ml/min. The aqueous buffer was prepared with 20 mM phosphoric acid and 40 mM tetrabutylammonium bromide, with the pH adjusted to 5.9. A sample volume of 5 μl was injected for each run, and analyte detection was monitored at a wavelength of 262 nm. For the qualitative identification of compounds, a Liquid Chromatography-Mass Spectrometry (LC–MS) approach was employed. The analysis utilized an Agilent 1290 UPLC system coupled to an Agilent QTOF 6550 mass spectrometer. Samples (5 μl) were injected onto a Waters BEH C18 column (2.1 × 100 mm, 1.7 μm particle size) and eluted at a flow rate of 0.4 ml/min. The mass spectrometer was equipped with an Electrospray Ionization (ESI) source operating with a capillary voltage of 3200 V. The sheath gas temperature was maintained at 350°C with a flow rate of 12 l/min.

### RNAi and overexpression of *PnSUS1* in *P. notoginseng*

Overexpression and RNAi of *PnSUS1 of A. tumefaciens* was cultivated in an MGL liquid medium until OD_600_ reached between 0.6 and 0.8, and then, the cultures of *A. tumefaciens* were centrifuged at 9000 *g* for 10 min and resuspended in the infiltration buffer (10 mM MgCl_2_, 10 mM MES, 200 mM acetosyringone; pH 5.6). Subsequently, WT *P. notoginseng* cells were grown on an MS medium supplemented with 40 mg l^−1^ acetosyringone for a duration of three days. Then, they were transferred into the *A. tumefaciens* suspension to create transgenic cell lines. Following the removal of *A. tumefaciens* using sterile water containing 400 mg l^−1^ cefotaxime, the transgenic cells were placed on an MS agar medium containing the same concentration of cefotaxime for 15 days. Finally, positive transgenic cell lines were selected using an MS medium containing 50 mg l^−1^ kanamycin sulfate. The notoginsenosides contents and gene expression were determined as described above.

### Subcellular localization

For transient expression assays to determine subcellular localization, *A. tumefaciens* strains harboring the EGFP fusion constructs were grown overnight in LB liquid medium. The bacterial cells were subsequently harvested by centrifugation, washed, and resuspended in an infiltration buffer (10 mM MgCl₂, 10 mM MES, pH 5.6) containing 150 μM acetosyringone. The final optical density of the bacterial suspension was adjusted to an OD_600_ of 0.8. This suspension was then infiltrated into the abaxial surface of 4- to 6-week-old *N. benthamiana* leaves using a needleless syringe. At 3 days post-infiltration, EGFP fluorescence in the epidermal cells was visualized using a NIKON AXR with NSPARC confocal laser scanning microscope.

### Transient overexpression in leaves of *P. notoginseng*

For transient gene overexpression assays, *A. tumefaciens* cultures were prepared as previously described, then resuspended in infiltration buffer to a final OD_600_ of 0.8. To perform the assay, the *Agrobacterium* suspension harboring the target gene construct was infiltrated into one half of a mature *P. notoginseng* leaf lamina. As a negative control, the opposite half of the same leaf, separated by the midvein, was infiltrated with an *Agrobacterium* strain containing the empty pCAMBIA1300 vector. Following infiltration, the plants were incubated in darkness for 24 h and then transferred to a controlled environment with a 16-h light/8-h dark photoperiod. At 5 dpi, leaf discs were harvested from both the experimental and control regions for subsequent metabolite profiling by HPLC.

### Dual-luciferase assays

To quantitatively assess transcription factor activity, a dual-luciferase reporter assay was conducted in *N. benthamiana* leaves. *A. tumefaciens* (strain GV3101) was separately transformed with an effector construct (containing the PnWRKY38 transcription factor) and a reporter construct (containing the target promoter fused to the Firefly luciferase gene, LUC). Cultures for each construct were grown, harvested, and resuspended in infiltration buffer. The effector and reporter suspensions were then mixed at a 10:1 (v/v) ratio and co-infiltrated into leaves. The Renilla luciferase (REN) gene, under the control of a constitutive 35S promoter, was included as an internal control for normalization. At 3 days post-infiltration, total protein was extracted from leaf tissue, and the luminescence from both Firefly (LUC) and Renilla (REN) luciferases was quantified using a Dual-Luciferase Reporter Assay System (Yeasen, Shanghai). The final transcriptional activity was expressed as the ratio of LUC to REN activity.

### Y1H assays

The bait plasmid, containing the *PnSUS1* promoter sequence cloned into pAbAi, was transformed into the Y1HGold yeast strain. To determine the basal expression level of the reporter gene, transformants were plated on SD/-Ura medium containing a gradient of Aureobasidin A (AbA) concentrations. The minimal AbA concentration that suppressed background growth (200 ng/ml) was used for all subsequent interaction assays. The prey plasmid, containing the *PnWRKY38* coding sequence cloned into pGADT7, was transformed into the Y1HGold strain already harboring the bait plasmid (pAbAi-PnSUS1). Co-transformants were selected and plated on high-stringency SD/-Leu/-Ura medium supplemented with 200 ng/ml AbA. Robust yeast growth on this selective medium was interpreted as a positive interaction between the PnWRKY38 protein and the *PnSUS1* promoter.

### Triterpene extraction and analysis

For sample preparation, the material was first dried at 55°C and ground into a fine powder. An aliquot of the powder (0.5 g) was suspended in 10 ml of methanol and subjected to ultrasonic-assisted extraction for 45 min. The resulting suspension was clarified by centrifugation at 8000 × *g* for 15 min, and the supernatant was collected and filtered through a 0.45 μm nylon membrane filter prior to analysis. Chromatographic analysis was performed on a Shimadzu HPLC system equipped with an SPD-M20A photodiode array (PDA) detector. The separation was conducted on a YMC-Pack Pro C-18 RS column (150 × 2.0 mm, 5 μm) maintained at 40°C. The mobile phase consisted of water (A) and acetonitrile (B) delivered at a flow rate of 0.5 ml/min. The gradient elution program was as follows: 0–5 min, 25%–35% B; 5–10 min, 35%–55% B; 10–15 min, 55%–70% B; 15–20 min, 70%–90% B; 20–30 min, 90%–100% B; 30–35 min, 100%–75% B; 35–40 min, 75%–25% B; followed by an isocratic hold at 25% B from 40 to 50 min for column re-equilibration.

### Statistical analysis

All experiments were conducted with a minimum of three replicates, and the results are presented as the mean ± SD. Statistical analyses were carried out using GraphPad Prism 7 software. Differences between groups were evaluated using two-tailed Student’s t-tests or Duncan’s post hoc analysis following ANOVA. *P*-value of less than 0.05 was deemed statistically significant (^*^P < 0.05; ^**^P < 0.01).

## Supplementary Material

Web_Material_uhag012

## Data Availability

All data supporting the results of this study are presented in the manuscript, including Supplementary information. The datasets generated or analyzed during the current study are available from the corresponding author on reasonable request.
