# Supplementary material for: PnWRKY38-PnSUS1 axis regulates the biosynthesis of Panax notoginseng saponins
Source: Hortic Res. 2026 Jan 13;13(4):uhag012. doi: 10.1093/hr/uhag012 (PMC13095356; doi:10.1093/hr/uhag012)
Supplement: Web_Material_uhag012 [file web_material_uhag012.zip › Supplementary files.docx]

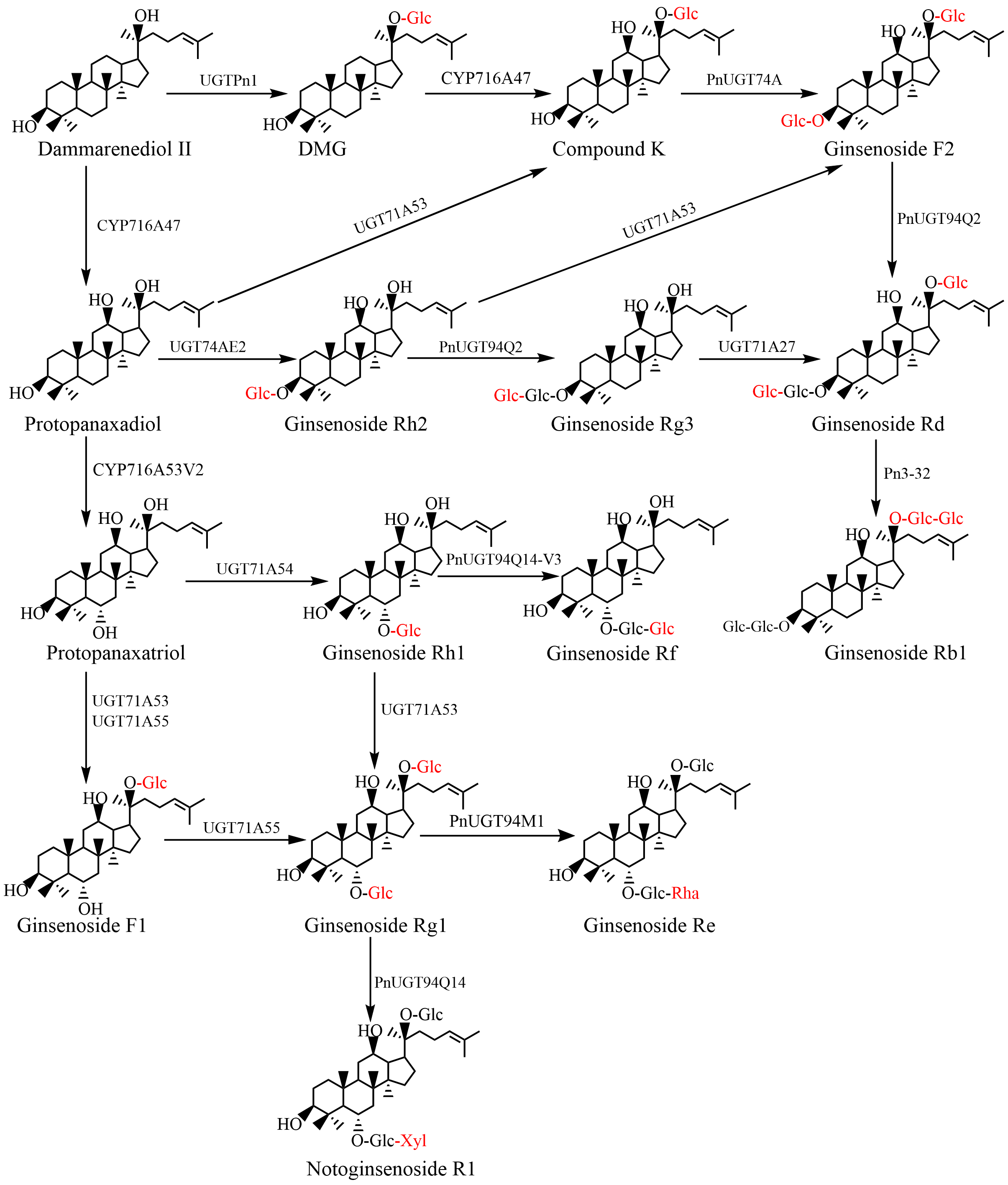


**Fig. S1 Glycosylation modification process in the synthesis of notoginsenosides.** The red group indicates the addition effect under the action of the relevant UGT.


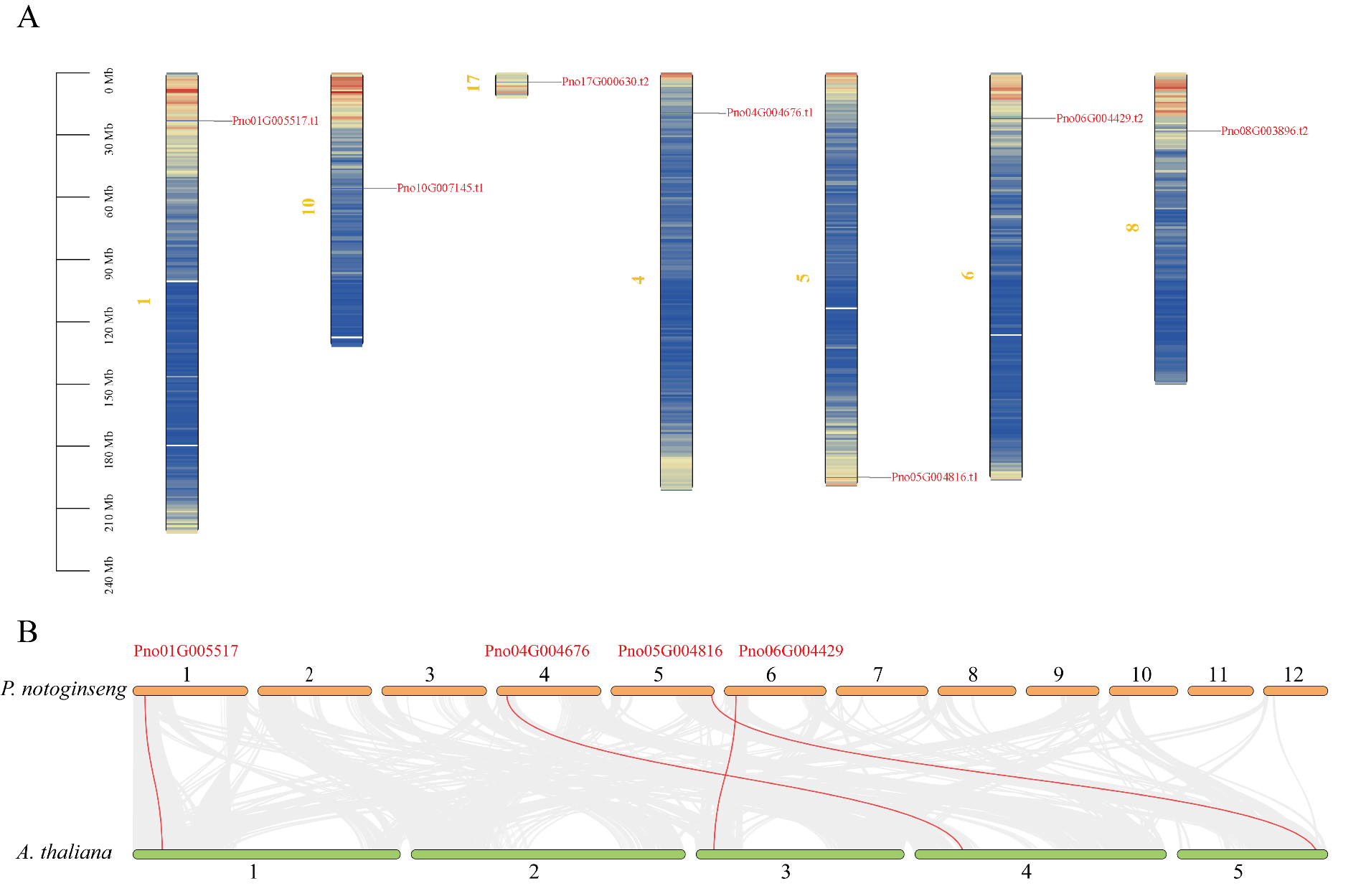


**Fig. S2 Identiﬁcation of *PNSUS1* from the *P. notoginseng* genome.** (A) Chromosomal localization analysis of *PnSUS*. (B) Collinearity analysis of PnSUS gene.

**Fig. S3 HPLC analysis of the enzymatic reaction products of PnSUS2.**


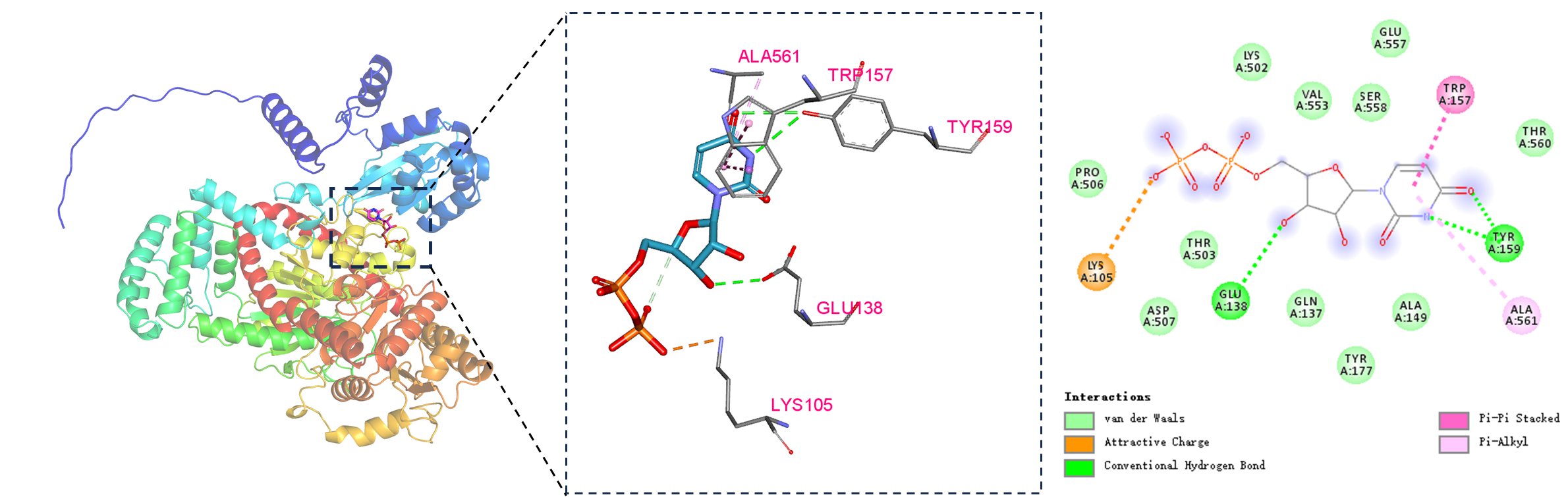


**Fig. S4 Molecular docking of PnSUS1 and UDP.**

**Fig. S5** **The expression levels of UGTs in the *P. notoginseng* cells with overexpression and RNAi of *PnSUS1***. A, The expression levels of UGTs in the *P. notoginseng* cells with overexpression of *PnSUS1*. B, The expression levels of UGTs in the *P. notoginseng* cells with RNAi of *PnSUS1*. The experiment was conducted three times, and error bars reflect the standard deviation (SD) from three independent biological replicates. Asterisks show statistically significant differences relative to WT. Statistical analysis was performed by one-way ANOVA with Duncan’s multiple range tests to separate means. ***P* < 0.01, **P* < 0.05.

**Fig. S6 Nine transcription factors and yeast one-hybrid experiments with the PnSUS1 promoter.**


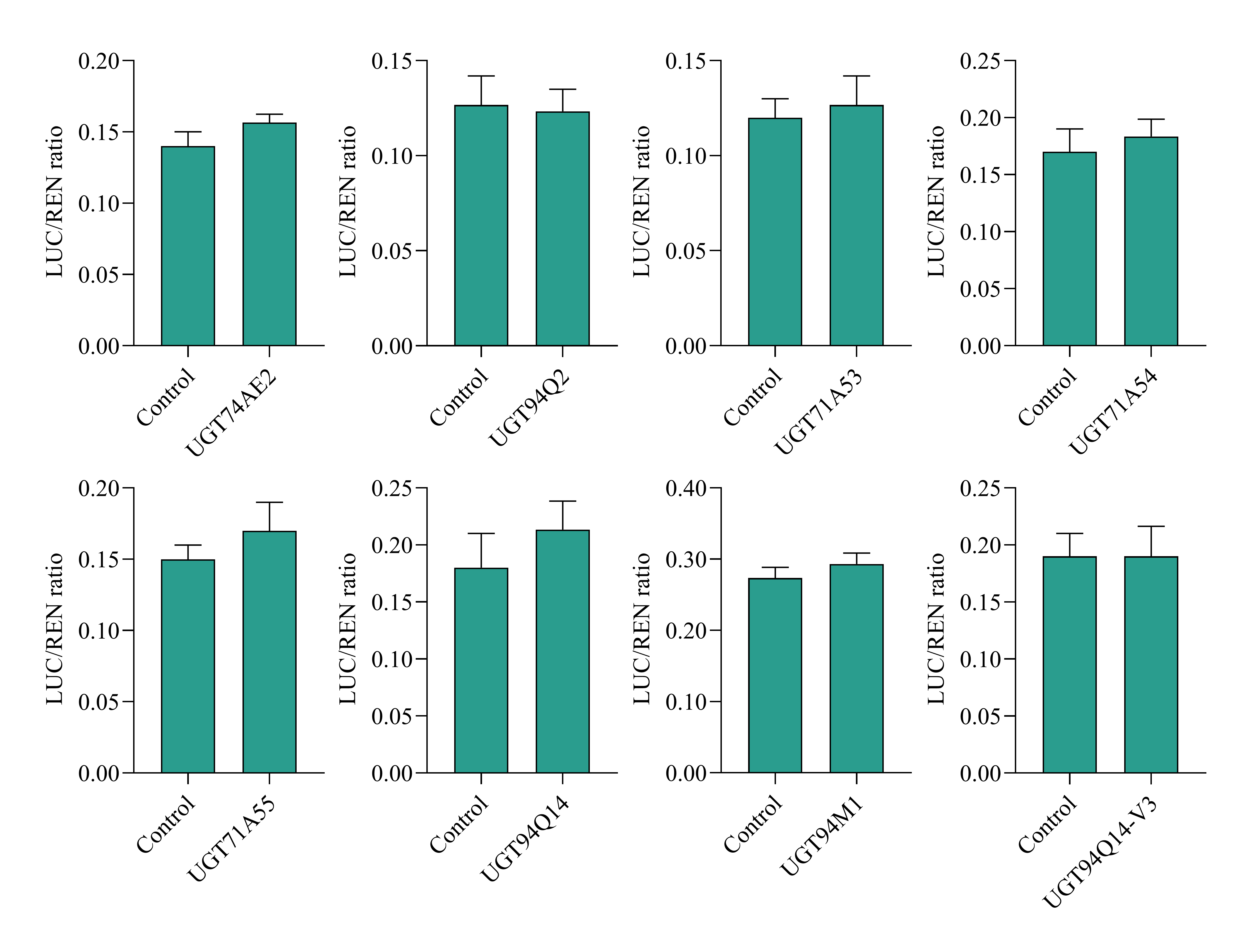


**Fig. S7 The LUC/REN ratio represents the relative activity of PnWRKY38 for different promoters of UGTs in *N. benthamiana*.** The experiment was conducted three times, and error bars reflect the standard deviation (SD) from three independent biological replicates. Statistical significance was determined using the Student’s t-test.

**Fig. S8 The content of monomer saponin in the co-expressed *PnSUS1* and *PnWRKY38* *P. notoginseng* cells.**
